# Characterization of the complete mitochondrial genome of the lung fluke, Paragonimus kellicotti

**DOI:** 10.1080/23802359.2020.1724669

**Published:** 2020-02-04

We, the Editor and Publisher of *Mitochondrial DNA Part B: Resources*, have retracted the following article:

Tao Wang, Yunfang Wang, Fengqiang Xu, Xia Li, Rui Qu, Lin Song, Yongping Tang & Peilin Lin (2018). Characterization of the complete mitochondrial genome of the lung fluke, Paragonimus kellicotti. *Mitochondrial DNA Part B: Resources*, 3:2, 715–716 https://doi.org/10.1080/23802359.2018.1483763

The findings reported in this article have been found to be unreliable and the methods described unsound. The data reported in the article was not created by the authors and the original source of the sequence was not acknowledged.

We have been informed in our decision-making by our policy on publishing ethics and integrity and the COPE guidelines on retractions.

The retracted article will remain online to maintain the scholarly record, but it will be digitally watermarked on each page as “Retracted”.

